# Immune reconstitution efficacy and associated risk factors for immunological non-response in people living with HIV during long-term antiretroviral therapy: a single-center retrospective cohort study at a tertiary care hospital in China

**DOI:** 10.3389/fimmu.2026.1776587

**Published:** 2026-03-13

**Authors:** Rui Li, Lifeng Liu, Ran Wang, Yuanyi Zhai, Yiming Ren, Wei Hua, Yang Zhang, Lijun Sun, Lili Dai

**Affiliations:** 1 Center for Infectious Diseases, Beijing Youan Hospital, Capital Medical University, Beijing, China; 2 Beijing Key Laboratory for HIV/AIDS Research, Sino-French Joint Laboratory for HIV/AIDS Research, Clinical and Research Center for Infectious Diseases, Beijing Youan Hospital, Capital Medical University, Beijing, China

**Keywords:** antiretroviral therapy, CD4/CD8 ratio, cohort study, HIV, immune reconstitution, immunological non-response

## Abstract

Despite sustained virological suppression under antiretroviral therapy (ART), a substantial proportion of people living with HIV (PLWH) fail to achieve adequate immune reconstitution. However, long-term real-world evidence characterizing immune recovery trajectories and determinants of immunological non-response (INR) has not yet been fully characterized, particularly in Asian populations. In this retrospective cohort study, we analyzed longitudinal immunological data from 8,637 PLWH who initiated ART between 2005 and 2019 at a large HIV treatment center in China and achieved sustained viral suppression. CD4 counts, CD8 counts, and CD4/CD8 ratios were assessed longitudinally to characterize immune reconstitution patterns, and Kaplan–Meier analyses and logistic regression models were used to evaluate factors associated with immune recovery and INR. We observed a biphasic pattern of immune reconstitution, with rapid early increases in CD4 counts and CD4/CD8 ratios followed by slower, sustained improvements over long-term ART. Nevertheless, normalization of the CD4/CD8 ratio occurred less frequently than CD4 count recovery, indicating persistent immune dysregulation in a subset of patients despite virological control. Lower baseline CD4 count and older age were the strongest predictors of incomplete immune reconstitution, with individuals initiating ART at advanced stages of immunodeficiency showing markedly reduced probability and slower tempo of immune normalization. These findings highlight the heterogeneity of immune recovery under long-term ART and underscore the importance of incorporating baseline immune status and age into risk stratification strategies. Longitudinal assessment of both CD4 counts and CD4/CD8 ratios may provide complementary insights into immune restoration and inform individualized monitoring and management of PLWH receiving ART.

## 1 Introduction

The introduction of ART has achieved durable viral suppression and reduced AIDS-related morbidity (1, 2). Improved virological control has been associated with immune recovery becoming a major treatment objective, and CD4 counts and the CD4/CD8 ratio are widely used to assess reconstitution in PLWH (3). However, recovery remains heterogeneous under ART (4). Prior longitudinal cohorts have suggested that immune reconstitution may follow nonlinear trajectories, with faster gains after treatment initiation and slower improvements during long-term therapy, but long-term patterns and variability across baseline immune levels are not well defined, particularly in real-world settings (5, 6). Importantly, previous studies have shown that recovery of CD4 counts does not necessarily occur in parallel with normalization of the CD4/CD8 ratio during long-term ART, suggesting that restoration of absolute CD4 levels may not fully reflect recovery of immune homeostasis.

Although most individuals experience immunological improvement after ART, a subset of PLWH still exhibit incomplete immune reconstitution despite sustained viral suppression. Approximately 10–40% of PLWH fail to achieve expected CD4 recovery or normalization of the CD4/CD8 ratio and are classified as immunological non-responders (INRs) (4, 7), a condition that has been associated with increased risks of opportunistic infections, non-AIDS comorbidities, and all-cause mortality (8). Large cohort studies from Europe and North America, including the Collaboration of Observational HIV Epidemiological Research Europe (COHERE), NA-ACCORD, and ICONA, have consistently demonstrated that baseline CD4 level is a major determinant of long-term immune reconstitution. Even under sustained virological suppression, patients initiating ART with low baseline CD4 counts often experience persistently limited immune recovery (9–11). Although longitudinal cohorts have evaluated immune recovery across baseline immunological strata, most evidence derives from Western populations, and long-term real-world data from Asian PLWH remain less well characterized and warrant further characterization (12). Given substantial regional differences in HIV epidemiology, transmission routes, timing of diagnosis, treatment initiation, and patterns of coinfections, immune recovery trajectories and long-term outcomes may differ considerably across populations.

To further contribute evidence in this area, we conducted a large single-center retrospective longitudinal cohort study based on a real-world population of PLWH in Beijing, China. Accordingly, this study aimed to systematically characterize long-term immune reconstitution under sustained virological suppression, with a particular focus on the dynamic trajectories of CD4 counts and CD4/CD8 ratio. Specifically, this study aimed to (1) describe the long-term dynamic changes in CD4 counts and CD4/CD8 ratio following ART across different baseline CD4 strata; (2) compare the cumulative probability of immune reconstitution among individuals with different baseline immune statuses, while evaluating the complementary value of the CD4/CD8 ratio in predicting long-term immune outcomes; and (3) identify factors associated with INRs.

## 2 Methods

### 2.1 Study design and population

This real-world retrospective cohort study utilized data from the STD/AIDS Clinical Diagnosis and Treatment Center of Beijing Youan Hospital, Capital Medical University, and included treatment-naive adults living with HIV who initiated antiretroviral therapy between January 1, 2005 and June 30, 2019. HIV infection was diagnosed by enzyme-linked immunosorbent assay (ELISA) and confirmed by Western blot or HIV-1 RNA nucleic acid amplification testing according to national guidelines (13). The inclusion criteria were: (1) age ≥18 years; (2) confirmed HIV infection as described above; (3) treatment-naïve PLWH who initiated ART for the first time between January 1, 2005 and June 30, 2019; (4) achieved virological suppression (HIV-1 RNA <20 copies/mL) for at least 3 years after ART initiation; (5) had available baseline CD4 count and CD4/CD8 ratio data. The exclusion criteria included: (1) those lacking essential information, such as baseline CD4 counts; (2) those without any follow-up records; (3) pregnancy at baseline. The study was approved by the Ethics Committee of Beijing Youan Hospital.

### 2.2 Procedure

This study analyzed long-term follow-up data from PLWH who initiated treatment between 2005 and 2019. The observation period extended from ART commencement until the earliest occurrence of: loss to follow-up, death, transfer-out, treatment discontinuation, or the last follow-up before December 31, 2021. Baseline assessments included plasma viral load, CD4 counts, CD4/CD8 ratio. General patient information, including age, ethnicity, education level, occupation, marital status, and route of infection, was also collected. After ART initiation, plasma viral load was measured at baseline, and at 3, 6, and 12 months, followed by annual assessments thereafter. CD4 count and CD4/CD8 ratio were evaluated at the same time points.

### 2.3 Definitions

In this study, INRs were defined as participants receiving ART ≥ 4 years, maintaining virological suppression ≥ 3 years, yet persistently showing CD4 counts < 350 cells/µL after exclusion of other causes of immunodeficiency (14, 15). Immune responses (IRs) was defined as achieving a CD4 count ≥500 cells/µL under sustained viral suppression, confirmed by at least two consecutive measurements during ART. Virological suppression was defined as plasma HIV-RNA < 50 copies/mL and virological failure as HIV-RNA > 200 copies/mL persisting ≥ 24 weeks after ART initiation (16).

### 2.4 Statistical analysis

Descriptive statistics summarized baseline characteristics, with categorical variables expressed as frequencies and percentages. Age, which showed an approximately symmetric distribution, was presented as mean ± standard deviation (SD). Skewed variables, including CD4 count, CD4/CD8 ratio, viral load, and time intervals, were expressed as median and interquartile range (IQR). To characterize longitudinal trajectories of CD4 count and CD4/CD8 ratio after ART initiation, generalized estimating equation (GEE) models were applied. Kaplan–Meier analysis estimated cumulative probabilities of immune reconstitution across baseline CD4 categories. Concordance between CD4 normalization and CD4/CD8 ratio normalization at predefined time points was evaluated using 2×2 contingency analyses with Cohen’s kappa statistics and McNemar’s test. Univariate and multivariate logistic regression models identified independent predictors. Multicollinearity among predictive variables was assessed using the variance inflation factor (VIF). Analyses were performed using Python 3.10 and SPSS 30.0, with statistical significance defined as *p* < 0.05.

## 3 Results

### 3.1 Baseline characteristics of the study population

**Figure 1:**
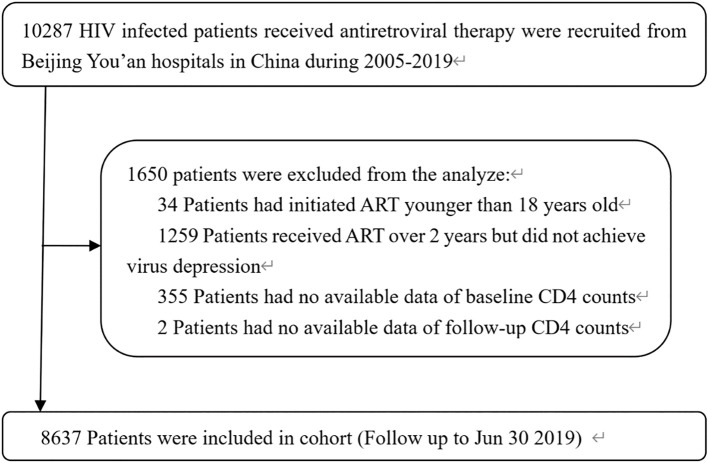
HIV-1-infected patients who initiated ART between January 1, 2005, and June 30, 2019. Flow chart showing the selection of PLWH who initiated ART between January 1, 2005 and June 30, 2019. Eligible participants were adults aged ≥18 years with confirmed HIV-1 infection and ≥3 years of sustained viral suppression. Patients lacking baseline CD4 data, without follow-up records, or with pregnancy at baseline were excluded. ART, antiretroviral therapy; PLWH, people living with HIV.

A total of 8,637 PLWH were included (**Figure 1**), with a median follow-up of 4.67 years (IQR 3.17–6.13). Baseline characteristics are summarized in (**Table 1**). Among the participants, 8,296 (96.05%) were male, of whom 7,525 (87.13%) were infected with HIV through men who have sex with men (MSM). The median age was 33.4 years, and the median time from HIV diagnosis to ART initiation was 34 (IQR: 15-134) days. The median baseline CD4 counts was 303 cells/µL (IQR 194.27-423.00), and the median CD4/CD8 ratio was 0.29 (IQR 0.18-0.43). The median viral load was 15,928 copies/mL (IQR 4,310-57,632). Approximately 798(9.30%) were classified as WHO stage III–IV. A small proportion were co-infected with hepatitis virus infection at the time of HIV diagnosis (HBV: 387 [4.48%]; HCV: 139 [1.61%]). At ART initiation, most participants (7,429 [80.46%]) received treatment regimens of two nucleoside reverse transcriptase inhibitors (NRTIs) with one non-NRTI (NNRTI).

**Table 1:** Baseline features of HIV-1-infected patients.

Characteristics	Value
Total patients, n	8,637
Sex, n (%)	
Male	8,296 (96.05)
Female	341 (3.95)
HIV transmission route, n (%)	
Blood transfusion	56 (0.65)
MSM	7,525 (87.13)
Heterosexual	616 (7.13)
Injection drug use	67 (0.78)
Others/Unknown	373 (4.32)
WHO clinical stage, n (%)	
III-IV	798 (9.25)
Co-infection, n (%)	
HBV infection	387 (4.48)
HCV infection	139 (1.61)
Days from HIV diagnosis to ART start, Median (IQR)	33.00 (15.00-134.00)
Age at diagnosis of HIV (years), mean ± SD	33.43 ± 10.30
Age at start of ART (years), n (%)	
<30	3,787 (43.85)
30-49	4,106 (47.54)
>50	744 (8.61)
Calendar year of start of ART, n (%)	
<2010	198 (2.29)
2010-2014	2,842 (32.90)
≥2015	5,597 (64.80)
Duration of follow-up on ART (years), Median (IQR)	4.67 (3.17, 6.13)
Range of follow-up on ART (years), n (%)	
<5	4,790 (55.46)
5-10	3,624 (41.96)
≥10	223 (2.58)
CD4 count at start of ART (cells/µL), median (IQR)	303.00 (194.27, 423.00)
CD4 count at start of ART, cells/μL, n (%)	
<50	697 (8.07)
50-199	1,541 (17.84)
200-349	3,059 (35.42)
350-499	2,043 (23.65)
≥500	1,297 (15.02)
CD4/CD8 ratio at start of ART, median (IQR)	0.29 (0.18, 0.43)
CD4/CD8 ratio at start of ART, n (%)	
<0.30	4,423 (51.21)
0.30-0.49	2,609 (30.21)
0.50-0.99	1,307 (15.13)
≥1.00	136 (1.57)
Viral load at start of ART (copies/ml) Median (IQR)	15,928.0 (4,310.5, 57,632.25)
Viral load at start of ART (copies/mL), n (%)	
≤5000	1,929 (27.46)
>5000, ≤30000	2,490 (35.44)
>30000, <100000	1,417 (20.17)
>100000, ≤500000	935 (13.31)
>500000	255 (3.63)
ART regimen at start of ART	
2 NRTIs + EFV/NVP	7,429 (80.46)
2 NRTIs + LPV/r or DRV/c	920 (9.99)
2 NRTIs+ BIC/DTG/RAL	178 (1.93)
LPV/r+3TC or DTG + 3TC or LPV/r+RAL	12 (0.13)
Others	98 (1.06)

Data are expressed as n (%) or median (IQR). ART, antiretroviral therapy; SD, standard deviation; IQR, interquartile range; HIV, human immunodeficiency virus; MSM, men who have sex with men; EFV, efavirenz; NVP, nevirapine; LPV/r, lopinavir/ritonavir; DRV/c, darunavir/cobicistat; BIC, bictegravir; DTG, dolutegravir; RAL, raltegravir; 3TC, lamivudine.

### 3.2 Dynamic changes in CD4 counts and the CD4/CD8 ratio after ART

**Figure 2:**
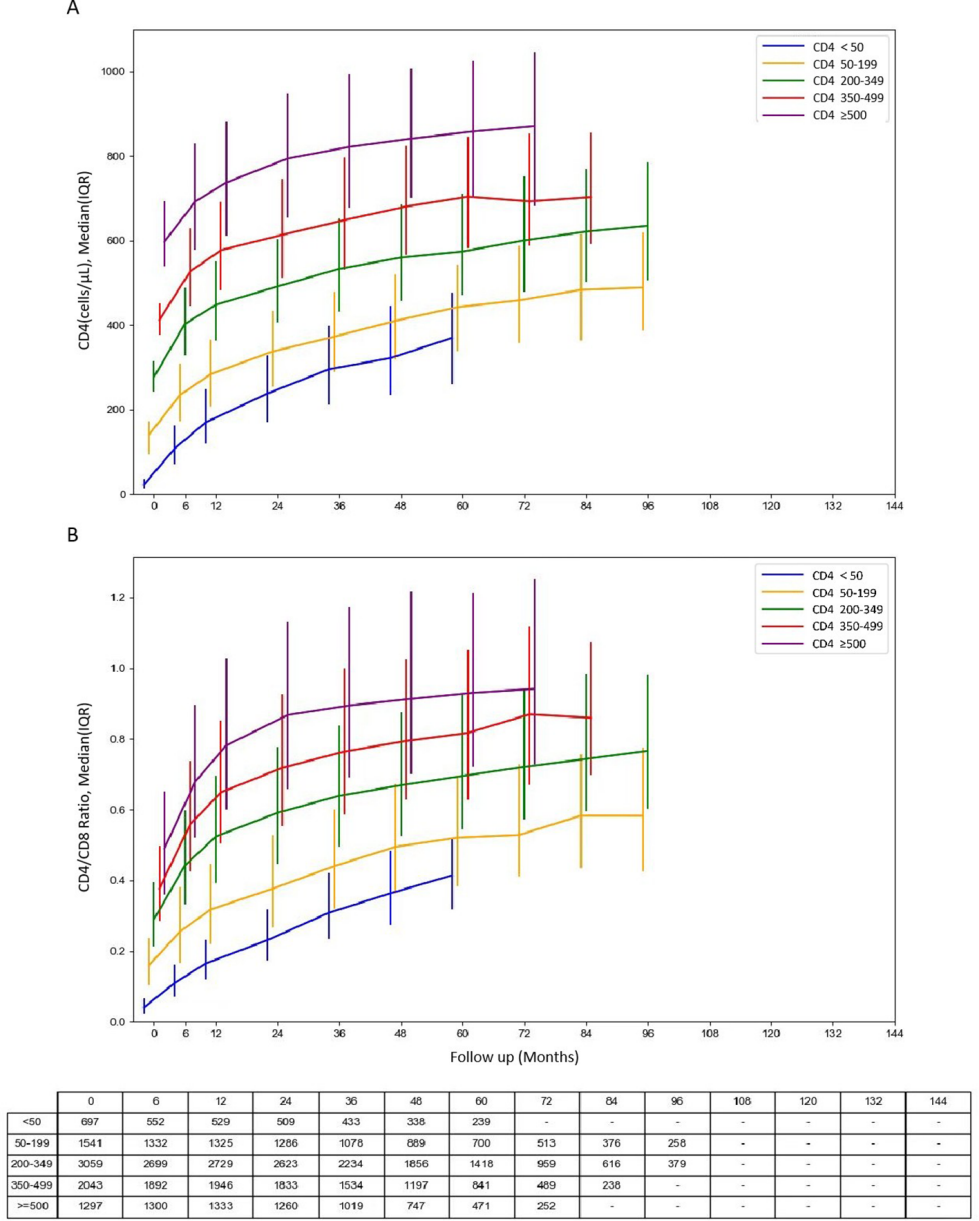
The dynamic trajectories of CD4 counts and CD4/CD8 ratio in HIV-1 patients based on five groups of baseline CD4 counts. **(A)** Dynamic changes in CD4 counts and **(B)** CD4/CD8 ratios among PLWH during long-term ART follow-up. Patients were stratified by baseline CD4 levels (<50, 50–199, 200–349, 350–499, and ≥500 cells/μL). Median values with interquartile ranges (IQRs) are shown for each time point. CD4 counts and CD4/CD8 ratios increased rapidly during the first 6 months after ART initiation, followed by slower recovery thereafter. Patients with baseline CD4 <200 cells/μL showed persistently lower immune recovery. ART, antiretroviral therapy; IQR, interquartile range.

CD4 counts increased substantially across all baseline CD4 strata during the first 6 months after ART initiation (**Figure 2A**). After 6 months of ART, the median CD4 counts in participants with five different baseline CD4 groups (<50, 50–199, 200–349, 350–499, ≥500 cells/µL) respectively reached 108.04 (71.44-162.0), 237.08 (173.92-310.0), 403.50 (328.89-488.96), 527.49 (444.80-630.28), and 692.63 (577.30-830.15) cells/µL (**Supplementary Table 1**). Participants with lower baseline CD4 counts showed larger absolute increases during the first 6 months of ART. However, their CD4 counts remained substantially lower than those of participants initiating ART at higher baseline levels. Notably, even after 5 years of ART, the median CD4 for those starting < 350 cells/µL remained below 500 cells/µL (**Supplementary Table 1**). To more precisely characterize longitudinal recovery patterns, GEE models were applied to evaluate changes in CD4 counts over time (**Table 2**; **Supplementary Table 3**). CD4 recovery followed a biphasic pattern, with a more rapid increase during the first 6 months after ART initiation, followed by a slower but sustained improvement thereafter. The rate of recovery differed significantly across baseline CD4 strata (time × baseline CD4 interaction, P<0.001). Although individuals with lower baseline CD4 counts demonstrated faster early recovery, the gap between baseline strata persisted over time, indicating that delayed ART initiation may have long-lasting consequences for immune restoration.

**Table 2:** GEE-estimated slopes of CD4 recovery before and after 6 months of ART, stratified by baseline CD4 level.

Baseline CD4 group (cells/µL)	0–6 months slope (log/month)	95% CI	>6 months slope (log/month)	95% CI
<50	0.351	(0.340, 0.363)	0.0149	(0.0139, 0.0159)
50–199	0.125	(0.121, 0.128)	0.008	(0.0076, 0.0083)
200–349	0.075	(0.073, 0.076)	0.0053	(0.0051, 0.0055)
350–499	0.049	(0.047, 0.051)	0.0044	(0.0041, 0.0047)
≥500	0.023	(0.020, 0.025)	0.0039	(0.0035, 0.0042)

Recovery rates were estimated using generalized estimating equation (GEE) models with log-transformed CD4 counts as the outcome. Time was modeled as a piecewise variable (0–6 months and >6 months after ART initiation). Baseline CD4 group and time × baseline CD4 interaction terms were included in the model. Robust standard errors were calculated. A significant interaction between time and baseline CD4 group was observed (P for interaction <0.001).

A similar pattern was observed for the CD4/CD8 ratio, the CD4/CD8 ratio increased during the early treatment phase across all baseline CD4 groups (**Figure 2B**). For example, among participants with baseline CD4 <50 cells/µL, the median CD4/CD8 ratio increased from 0.04 at baseline to 0.11 at 6 months and reached 0.41 at 5 years. Participants initiating ART at higher baseline CD4 levels achieved correspondingly higher CD4/CD8 ratios over time. However, despite prolonged viral suppression, most groups did not consistently attain a CD4/CD8 ratio ≥0.8 even after extended follow-up (**Supplementary Table 2**). Longitudinal modeling confirmed that CD4/CD8 recovery was also characterized by an early rapid phase followed by a gradual deceleration (**Table 3**; **Supplementary Table 4**), and recovery trajectories differed significantly according to baseline immune status (P for interaction <0.001). Collectively, these findings suggest that immune reconstitution after ART is dominated by early restoration, while long-term immune equilibrium remains strongly influenced by baseline CD4 levels at treatment initiation.

**Table 3:** GEE-estimated slopes of CD4/CD8 ratio recovery before and after 6 months of ART, stratified by baseline CD4 level.

Baseline CD4 group (cells/µL)	0–6 months slope (log/month)	95% CI	>6 months slope (log/month)	95% CI
<50	0.227	(0.216, 0.238)	0.0161	(0.0149, 0.0173)
50–199	0.105	(0.101, 0.109)	0.009	(0.0085, 0.0094)
200–349	0.089	(0.087, 0.091)	0.0065	(0.0062, 0.0067)
350–499	0.082	(0.080, 0.085)	0.0057	(0.0053, 0.0060)
≥500	0.071	(0.068, 0.074)	0.0056	(0.0052, 0.0060)

Recovery rates were estimated using generalized estimating equation (GEE) models with log-transformed CD4/CD8 ratio as the outcome. Time was modeled as a piecewise variable (0–6 months and >6 months after ART initiation). Baseline CD4 group and time × baseline CD4 interaction terms were included in the model. Robust standard errors were calculated. A significant interaction between time and baseline CD4 group was observed (P for interaction <0.001).

### 3.3 The cumulative probability of immune reconstitution after ART in patients with different baseline CD4 counts

**Figure 3:**
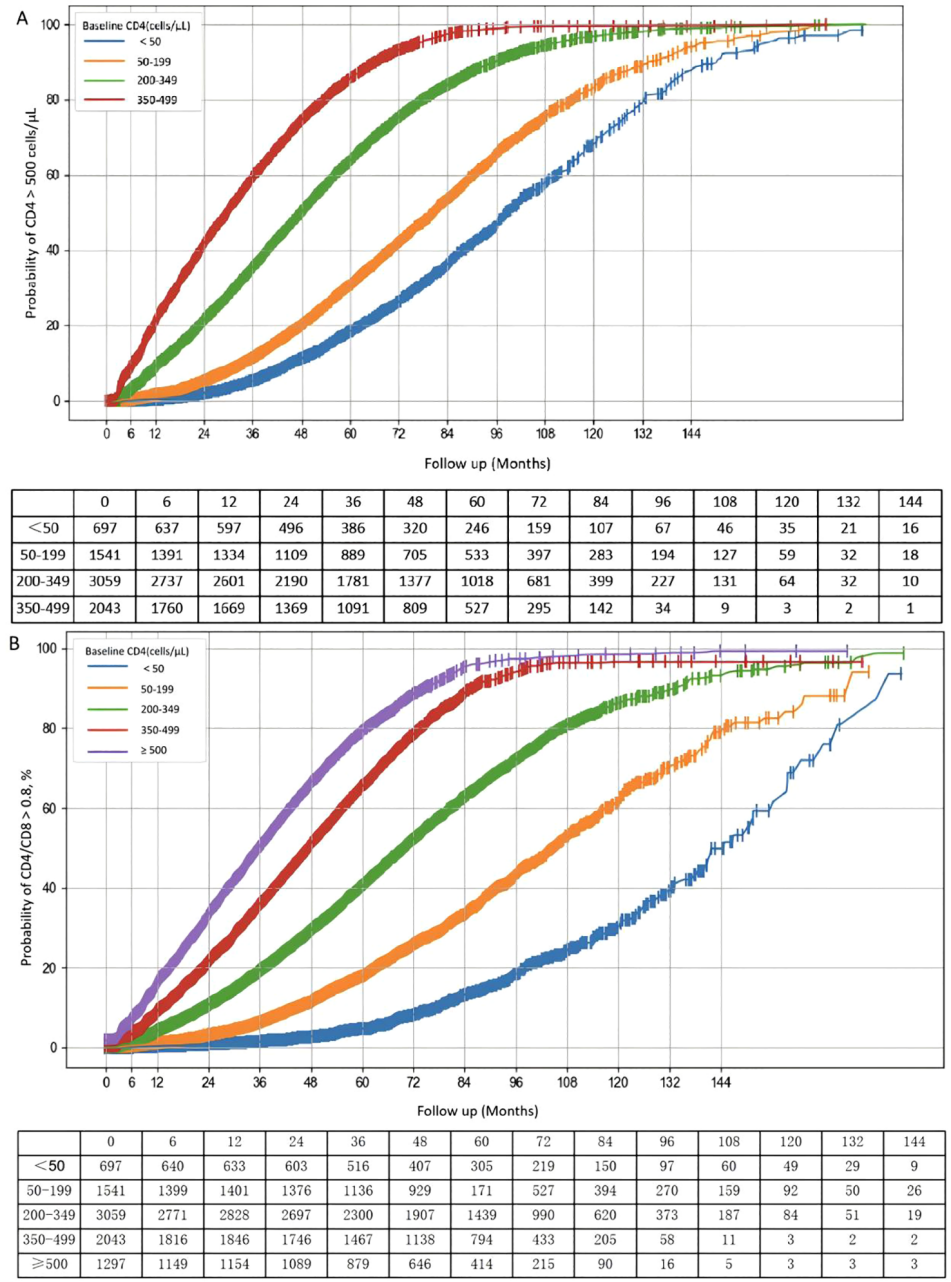
The cumulative probability of restoration of CD4 counts and CD4/CD8 ratio in PLWH after ART initiation. Kaplan–Meier curves showing the cumulative probability of achieving immune response (IR) under sustained viral suppression, stratified by baseline CD4 strata. IR was defined as achieving CD4 ≥500 cells/μL **(A)** or CD4/CD8 ratio ≥0.8 **(B)**, sustained for at least two consecutive follow-up measurements under viral suppression. Patients initiating ART with CD4 <200 cells/μL had a substantially lower probability of immune reconstitution during long-term treatment compared with those with higher baseline CD4 levels. IR, immune response; ART, antiretroviral therapy.

Kaplan–Meier analysis showed that the probability of achieving IR increased with baseline CD4. Within 3 years of ART, the probability of IR was 5.4%, 11.4%, 35.4%, and 59.4% for baseline CD4 < 50, 50–199, 200–349, and 350–499 cells/µL, respectively. After 10 years, these probabilities rose to 68.2%, 83.1%, 96.8%, and 99.6%. Overall, higher baseline CD4 counts were associated with faster and more complete immune recovery. A similar baseline-dependent pattern was observed for CD4/CD8 ratio normalization (**Figure 3**). However, distributional analyses at predefined follow-up time points indicated that CD4 normalization and CD4/CD8 ratio normalization were not fully concordant. Among participants who achieved CD4 ≥500 cells/µL, 63.50% at 12 months, 54.67% at 36 months, and 51.26% at 60 months after ART initiation still had a CD4/CD8 ratio <0.8, with 49.14% remaining below this threshold at the last follow-up (**Supplementary Table 5**).

**Figure 4:**
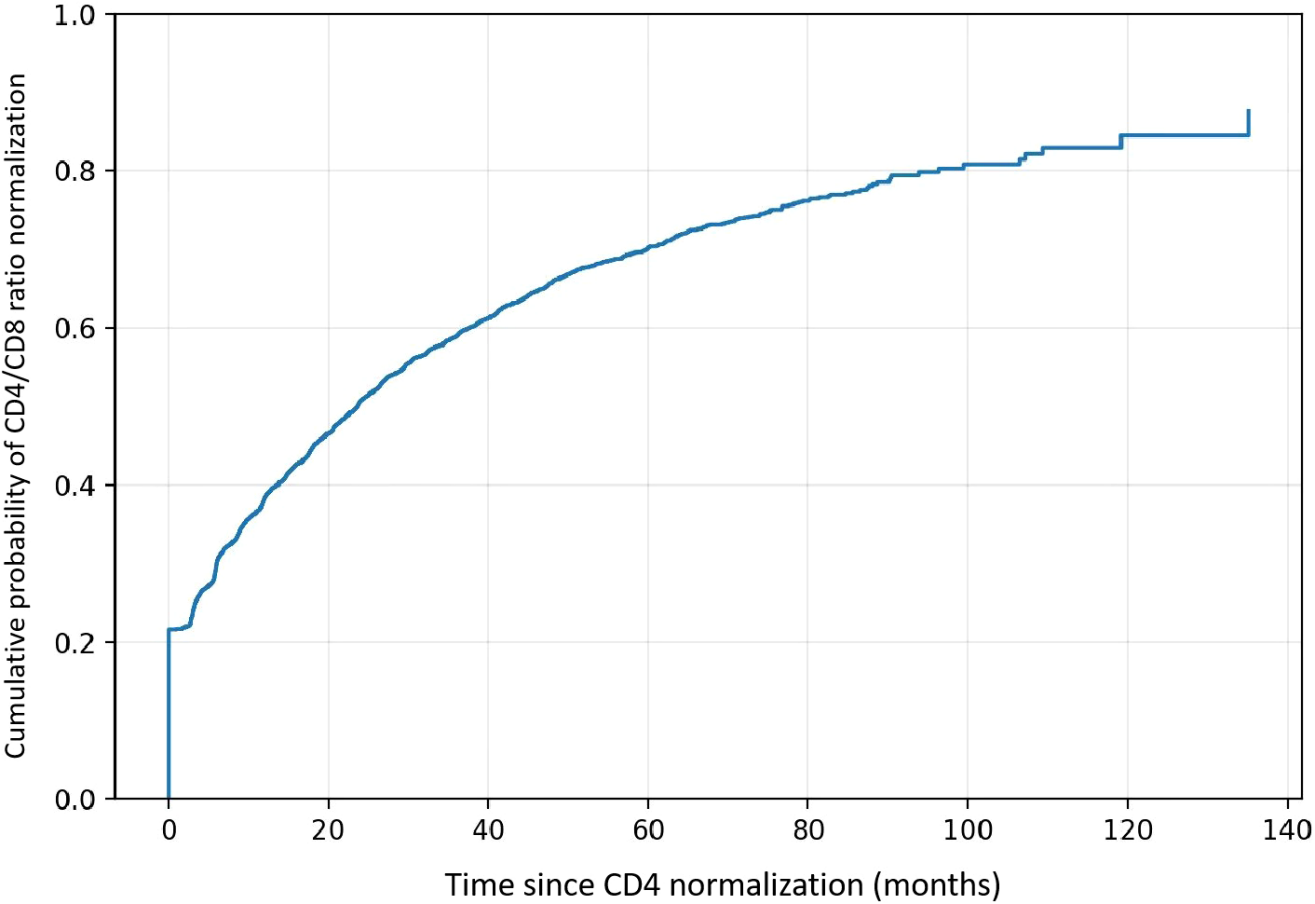
Time from CD4 normalization to CD4/CD8 ratio normalization. Kaplan–Meier curve showing the cumulative probability of achieving CD4/CD8 ≥0.8 after first attaining CD4 ≥500 cells/µL. Time zero was defined as the date of CD4 normalization. Participants not achieving CD4/CD8 ≥0.8 were censored at last follow-up.

To formally evaluate concordance between the two endpoints, 2×2 analyses were performed at predefined time points (**Table 4**; **Supplementary Table 6**). Agreement was consistently low to moderate (κ = 0.033–0.309), and McNemar’s tests demonstrated significant systematic differences at all time points (all P < 0.001), indicating that CD4 normalization occurred more frequently than CD4/CD8 ratio normalization. Further, among individuals who achieved CD4 ≥500, time-to-event analysis showed that CD4/CD8 ratio normalization often occurred later and remained incomplete in a substantial proportion of patients (**Figure 4**). Collectively, these findings indicate that CD4 recovery and CD4/CD8 ratio normalization represent related but distinct dimensions of immune reconstitution during long-term ART.

**Table 4:** Concordance between CD4 normalization and CD4/CD8 normalization at predefined time points.

Follow-up time	N	CD4 ≥500, n (%)	CD4/CD8 ≥0.8, n (%)	κ (Kappa)	McNemar P-value
36 months	5951	4230 (71.2%)	2406 (40.4%)	0.284	<0.001
60 months	3182	2556 (80.3%)	1595 (50.1%)	0.257	<0.001
120 months	189	162 (85.7%)	95 (50.3%)	0.033	<0.001
Final follow-up	8637	6677 (77.3%)	4256 (49.3%)	0.309	<0.001

CD4 normalization was defined as CD4 ≥500 cells/µL. CD4/CD8 ratio normalization was defined as CD4/CD8 ≥0.8. Agreement between the two endpoints was assessed using Cohen’s kappa statistic. McNemar’s test was used to evaluate systematic differences between paired proportions.

### 3.4 Factors affecting INRs in PLWH

**Figure 5:**
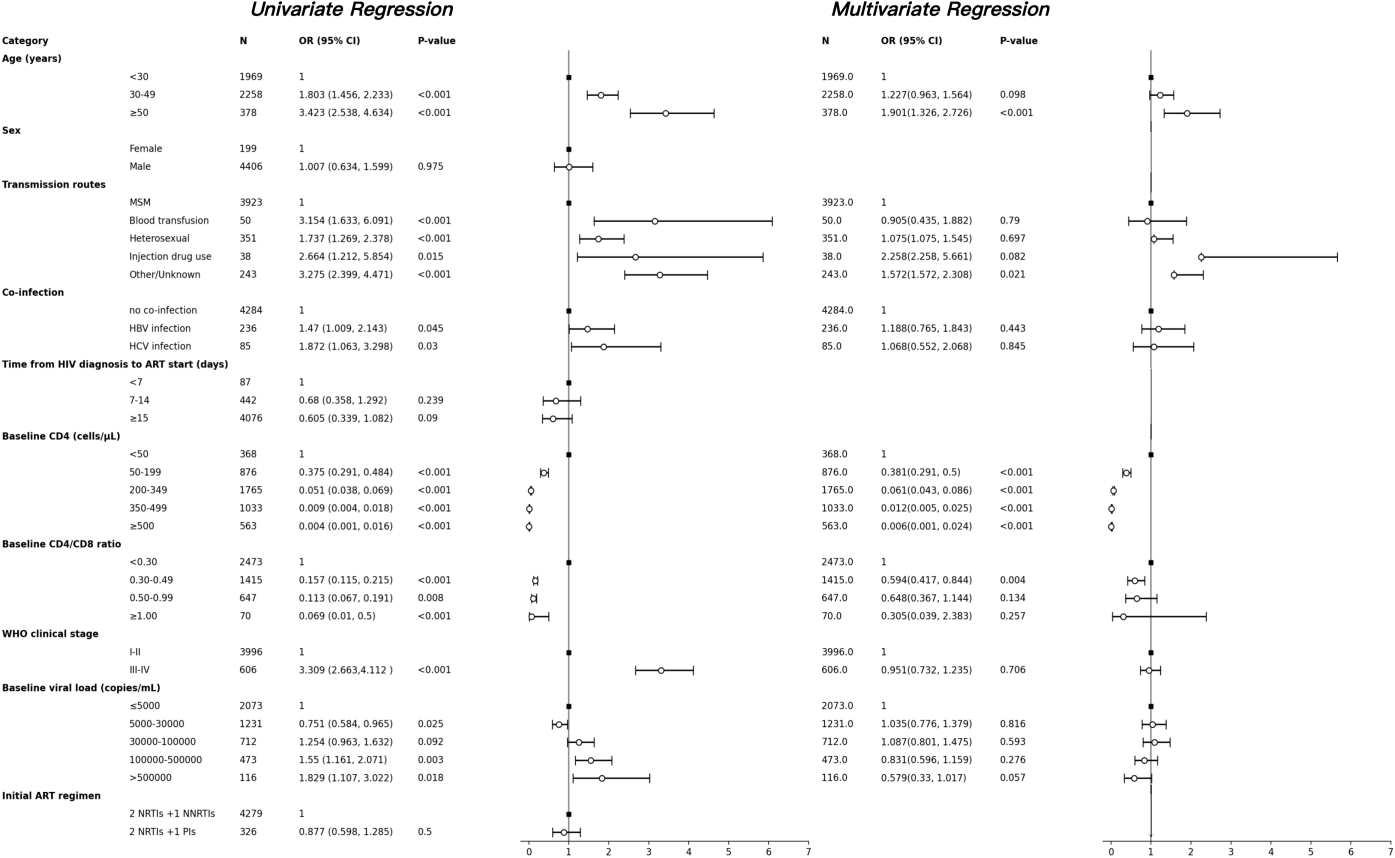
Factors associated with poor immune reconstitution identified by univariate and multivariate logistic regression analyses. Forest plot showing the odds ratios (ORs) and 95% confidence intervals (CIs) for clinical predictors of immunological non-response (INR) identified by univariate and multivariate logistic regression analyses. Independent risk factors include older age, lower baseline CD4 count. INR, immunological non-response; OR, odds ratio; CI, confidence interval; ART, antiretroviral therapy.

Among the 8,637 participants included in the analysis, 489 (5.7%) were classified as INRs. Several variables were independently associated with poor immune reconstitution: older age, heterosexual or blood transmission routes, HBV/HCV co-infection, lower baseline CD4 counts and CD4/CD8 ratio, advanced WHO stage, and higher baseline viral load. To assess potential multicollinearity among independent variables included in the multivariable logistic regression model, collinearity diagnostics were performed for candidate variables with P < 0.10 in the univariate analysis by calculating the VIF (**Supplementary Table 7**). All variables demonstrated VIF values below 5, indicating no evidence of significant multicollinearity; therefore, all candidate variables were retained in the multivariable logistic regression analysis. Age≥50 years (OR: 1.901, 95% CI: 1.326-2.726, P<0.001) remains a risk factor for failure of CD4 normalization. Using a baseline CD4<50 cells/µL as the reference group, the higher CD4 counts, the more favorable it is for immune reconstitution, 50-199 (OR:0.381,95% CI:0.291-0.500,P<0.001), 200-349 (OR:0.061,95% CI:0.043-0.086,P<0.001), 350-499 (OR:0.012,95% CI:0.005-0.025,P<0.001), ≥500 (OR:0.006,95% CI:0.001-0.024,P<0.001). Additionally, a baseline CD4/CD8 ratio of 0.30-0.49 (OR: 0.594, 95% CI: 0.417-0.844, P<0.001) was favorable for immune reconstitution in PLWH (**Figure 5**).

## 4 Discussion

In this large real-world cohort of 8,637 PLWH with long-term virological suppression, we found that immune reconstitution under ART followed heterogeneous and baseline-dependent trajectories (17, 18). CD4 counts and CD4/CD8 ratios increased rapidly during the early phase of treatment and improved slowly in the long term; however, individuals with lower baseline CD4 counts showed persistently limited recovery and a substantially lower probability of achieving immunological normalization (4, 19). Baseline CD4 count and age were the strongest predictors of immune outcome, while older age and lower baseline CD4 counts were consistently associated with INRs (20, 21). These findings support the incorporation of age and baseline immune status into long-term risk stratification frameworks for PLWH under ART (7). Moreover, they provide a clinical foundation for future investigations into the biological mechanisms underlying impaired immune recovery, including immunosenescence, thymic involution, and the limited regenerative capacity following advanced immune damage (22, 23). Importantly, normalization of the CD4/CD8 ratio occurred less frequently than CD4 count recovery, indicating that restoration of CD4 levels does not necessarily reflect recovery of immune homeostasis (1, 24).

This study provides several strengths in advancing the understanding of long-term immune reconstitution under ART. First, based on a large real-world cohort with an extended follow-up period, this study systematically characterized long-term immune reconstitution trajectories under sustained virological suppression. Second, we demonstrated a dissociation between recovery of CD4 counts and normalization of the CD4/CD8 ratio, showing that CD4 count normalization does not necessarily indicate restoration of immune homeostasis and that reliance on CD4 counts alone may overestimate immune reconstitution in a subset of patients. By providing long-term real-world evidence from an Asian population, our findings validate and extend observations from Western cohorts and support a more comprehensive evaluation of immune reconstitution in routine clinical practice.

The demographic and immunological characteristics of our cohort align with current global HIV epidemiological trends, particularly the predominance of men and the high proportion of MSM (87.13%) (25). This pattern may be associated with their sexual behavior patterns and social networks. The median baseline CD4 counts of 303 cells/μL indicates varying degrees of HIV-related immunodeficiency at treatment initiation (26). More than half of PLWH initiated ART with CD4 counts <350 cells/μL (52.3%), reflecting substantial immune impairment at baseline. Delayed treatment initiation likely constrains subsequent immune recovery, underscoring the need to strengthen early HIV screening and diagnosis, particularly among high-risk populations (27, 28).

Our longitudinal analysis revealed a clear two-phase pattern of immune reconstitution across baseline CD4 strata. CD4 counts and CD4/CD8 ratios increased rapidly within the first six months after ART initiation, likely reflecting intensive thymic output and peripheral expansion as viral replication was suppressed (17, 29). Thereafter, recovery entered a slower and more sustained phase, suggesting limited regenerative capacity following long-term immune damage (19). Individuals initiating ART with very low CD4 levels, particularly <350 cells/µL, rarely achieved CD4 normalization even after many years of treatment, indicating that a critical window for immune restoration may be lost once profound immunodeficiency has been established (30). This biphasic recovery pattern and its strong dependence on baseline immune status are consistent with findings from large Western cohort collaborations, including the COHERE and the ART-CC (9, 31). Across these studies, CD4/CD8 ratio recovery consistently lagged behind CD4 count restoration, reflecting persistent immune dysregulation despite durable viral suppression (32).

In Asian real-world settings, longitudinal studies have reported similar challenges in achieving complete immune restoration. A cohort study conducted in China highlighted incomplete CD4/CD8 ratio normalization under ART and emphasized the importance of early treatment initiation, but its primary focus was on metabolic alterations and long-term treatment safety, particularly among women (12). In contrast, another large national cohort emphasized mortality prediction using composite immune endpoints integrating CD4 count and CD4/CD8 ratio, whereas our study focuses on longitudinal immune reconstitution trajectories and risk stratification across baseline immune strata (6). By characterizing the dynamic constraints of immune recovery over extended follow-up, our findings provide complementary evidence to support individualized immune monitoring and inform subsequent analyses of factors associated with incomplete immune reconstitution (16, 33).

Kaplan–Meier analysis further demonstrated that baseline CD4 count is not only associated with short-term recovery but also determines the long-term probability and speed of immune normalization (34, 35). Patients initiating ART with higher CD4 levels at ART initiation showed a markedly greater chance of achieving immune reconstitution over time, whereas those with <200 cells/µL experienced prolonged and incomplete recovery trajectories (36). Consistent with findings from large international cohorts such as COHERE and the North American AIDS Cohort Collaboration on Research and Design (NA-ACCORD), our results confirm that baseline CD4 count at ART initiation remains a major determinant of long-term immune recovery despite sustained virological suppression. Importantly, CD4/CD8 ratio normalization occurs less frequently than CD4 count recovery, and a subset of patients with normalized CD4 counts continue to display persistently abnormal CD4/CD8 ratios during long-term follow-up (10). These findings suggest that numerical CD4 recovery does not necessarily imply full immune restoration. Compared with European cohorts, such as the Italian Cohort of Antiretroviral-Naïve Patients (ICONA), in which ART is more frequently initiated at relatively preserved CD4 levels and more complete immune recovery has been reported, the lower rates of CD4 and CD4/CD8 normalization observed in our cohort may reflect delayed treatment initiation and more advanced immune damage at baseline (11). Although prolonged ART gradually improves immune parameters, probability gaps across baseline CD4 strata persist for many years, suggesting structurally constrained immune restoration following late treatment initiation (37, 38). These findings align with recent international guidelines emphasizing immediate ART initiation and support the expansion of early HIV testing strategies in real-world care settings (39).

In addition to characterizing long-term immune trajectories, our study identified several key determinants of INRs in PLWH receiving ART. Older age significantly increased the likelihood of INR, consistent with age-associated thymic involution and reduced T-cell output that limit immune recovery (20, 40, 41). This observation is biologically plausible and consistent with prior NA-ACCORD and ART-CC large cohort analyses, highlighting immunosenescence as a key constraint on immune recovery (42). Baseline immunologic status remained the strongest predictor: individuals with very low CD4 counts at ART initiation were substantially less likely to achieve immune reconstitution, highlighting the importance of early HIV detection and treatment initiation (43). These findings further suggest that prolonged immune damage prior to ART initiation may predispose individuals to a persistent INR phenotype that is difficult to reverse, even under long-term virological suppression. Collectively, these findings emphasize the multifactorial nature of INR and underscore the need for enhanced monitoring and tailored clinical management in patients presenting with these risk profiles (24).

Several limitations should be acknowledged. The retrospective design may introduce inherent bias, and mechanistic data were unavailable. Further experimental studies are needed to elucidate these observations. Additionally, as the study population is exclusively drawn from Beijing, China, this introduces uncertainties regarding the applicability and generalizability of our findings to other populations or ethnic groups. Future multicenter studies incorporating immunological and molecular profiling are warranted.

In conclusion, immune reconstitution under long-term ART is highly heterogeneous and largely determined by baseline immune status. Early ART initiation substantially improves long-term recovery prospects, while delayed treatment leads to persistent immune deficits despite sustained viral suppression. The CD4/CD8 ratio provides complementary information on immune homeostasis and should be considered in long-term treatment assessment. These findings reinforce the importance of early HIV diagnosis, immediate ART initiation, and risk-stratified management to optimize treatment outcomes for PLWH.

## Data Availability

The raw data supporting the conclusions of this article will be made available by the authors, without undue reservation.
